# Human breast cancer associated fibroblasts exhibit subtype specific gene expression profiles

**DOI:** 10.1186/1755-8794-5-39

**Published:** 2012-09-06

**Authors:** Julia Tchou, Andrew V Kossenkov, Lisa Chang, Celine Satija, Meenhard Herlyn, Louise C Showe, Ellen Puré

**Affiliations:** 1 Department of Surgery, Division of Endocrine and Oncologic Surgery, Rena Rowan Breast Center, Abramson Cancer Center, Perelman School of Medicine of the University of Pennsylvania, Philadelphia, PA, 19104, USA; 2 The Wistar Institute, Philadelphia, PA, 19104, PA

## Abstract

**Background:**

Breast cancer is a heterogeneous disease for which prognosis and treatment strategies are largely governed by the receptor status (estrogen, progesterone and Her2) of the tumor cells. Gene expression profiling of whole breast tumors further stratifies breast cancer into several molecular subtypes which also co-segregate with the receptor status of the tumor cells. We postulated that cancer associated fibroblasts (CAFs) within the tumor stroma may exhibit subtype specific gene expression profiles and thus contribute to the biology of the disease in a subtype specific manner. Several studies have reported gene expression profile differences between CAFs and normal breast fibroblasts but in none of these studies were the results stratified based on tumor subtypes.

**Methods:**

To address whether gene expression in breast cancer associated fibroblasts varies between breast cancer subtypes, we compared the gene expression profiles of early passage primary CAFs isolated from twenty human breast cancer samples representing three main subtypes; seven ER+, seven triple negative (TNBC) and six Her2+.

**Results:**

We observed significant expression differences between CAFs derived from Her2+ breast cancer and CAFs from TNBC and ER + cancers, particularly in pathways associated with cytoskeleton and integrin signaling. In the case of Her2+ breast cancer, the signaling pathways found to be selectively up regulated in CAFs likely contribute to the enhanced migration of breast cancer cells in transwell assays and may contribute to the unfavorable prognosis of Her2+ breast cancer.

**Conclusions:**

These data demonstrate that in addition to the distinct molecular profiles that characterize the neoplastic cells, CAF gene expression is also differentially regulated in distinct subtypes of breast cancer.

## Background

Gene expression profiling of whole breast tumors has stratified breast cancer into several molecular subtypes that largely correlate with the expression status of three receptors in the tumor cells, namely estrogen (ER), progesterone (PR), and Her2-neu (Her2) [1,2]. The most common breast cancer subtype expresses either ER or PR but lacks Her2 expression. Breast cancers that do not express any of the 3 receptors, known as triple negative breast cancer (TNBC), and those that express Her2 (Her2+) are less common, comprising approximately 15% and 25% of all breast cancers respectively. Her2+ and TNBC have less favorable prognosis compared to ER + cancers [3,4]. How cancer cells acquire a specific molecular phenotype is uncertain. It has been postulated recently that the tumor stroma and the cancer cells may co-evolve to support the selection or enrichment of a specific cancer subtype [5].

Much of the earlier gene expression profile analyses of breast cancer were performed using RNA extracted from tumor samples comprised of at least 50% of tumor cells, with the tumor stromal cells being a minor but important component. As tumor cell survival and tumor progression are dependent on the tumor microenvironment, elucidating the symbiotic relationship between neoplastic cells and stromal cells is crucial to further our understanding of the pathogenesis of the disease [5-8]. This interdependency is reinforced by the recent identification of a stroma-derived gene signature that correlates with prognosis suggesting that the tumor stroma contributes significantly to the invasive and metastatic potential of tumor cells [9]. A unique breast cancer stroma signature has also been observed in women of African American descent compared to European American descent [10], while a stromal gene signature has been reported to predict response to chemotherapy [11]. These observations support the suggestion that intrinsic heterogeneities between the tumor stroma may correlate with patient-specific characteristics, prognosis, therapeutic response, and, perhaps, tumor subtypes. However, breast cancer subtype-specific differences have not yet been reported for the tumor stromal cells even though multiple studies have shown that the gene expression profiles of breast cancer associated fibroblasts (CAFs) are distinctly different from their normal counterparts. None of these prior studies had stratified their results based on tumor subtypes [12-16].

In this study, we isolated CAFs from twenty primary breast cancer samples representing three main subtypes (ER + (n = 7), TNBC (n = 7), Her2+ (n = 6)) and performed gene expression profile analyses on RNA isolated from these early passage CAFs. Subtype-specific gene expression profile differences were observed that distinguished CAFs derived from Her2+ cancers and TNBC and ER + cancers. Several genes, e.g. ITGA3, ITGA5, CFL1, and RHOA, that were found to be selectively up regulated in CAFs derived from Her2+ but not ER + or TNBC breast cancers are known to be involved with pathways associated with integrin and RhoA signaling suggesting that CAFs may contribute to the invasiveness of Her2+ breast cancer [17]. Migration of breast cancer cells,T47D, was significantly enhanced by CAFs derived from Her2+ breast cancer compared with ER + or TNBC. Our findings suggest that CAFs might contribute to the biology of the disease in a subtype-specific manner. Our findings are also consistent with the recently proposed tumor-stroma co-evolution hypothesis [5].

**Figure 1:**
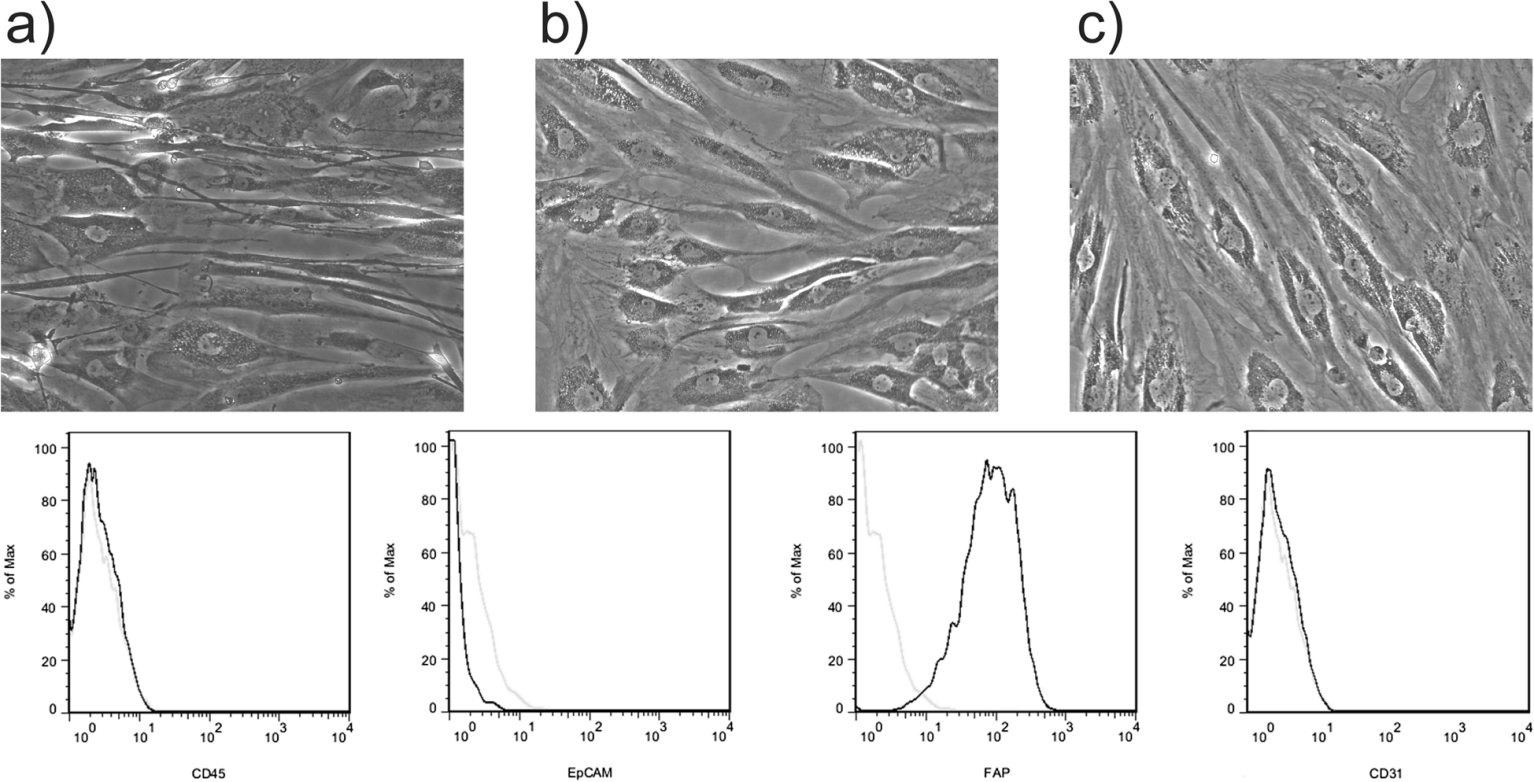
**qRT-PCR validation.** qPCR was used to validate microarray results for 6 genes found to be significantly different in either Her2+ vs ER+, Her2+ vs TNBC or ER + vs TNBC comparison in microarrays data. Expression for arrays and qPCR were normalized separately over average value across absolute expression for Her2 +, ER + and TNBC groups. Error bars represent standard error of mean for the group.

## Methods

### Patients and clinical characteristics of study cohort

Women with primary operable breast cancer undergoing breast surgery at the Hospital of the University of Pennsylvania were asked to participate in our tissue banking protocol approved by the institutional review board. Informed consent was obtained from all participants. Our study cohort included 20 women diagnosed with breast cancer between 2008 and 2011. Breast tumors were stratified into three subgroups according to receptor expression determined by immunohistochemistry (IHC) as described previously [18]: 1) ER + denotes breast cancer which expresses either ER or PR and lacks Her2 expression (n = 7); 2) TNBC denotes breast cancer that lacks expression of ER, PR, and Her2 (n = 7); and 3) Her2+ group (n = 6) denotes breast cancer which expresses Her2 as determined by IHC and/or fluorescence in situ hybridization with (n = 1) or without expression of ER or PR (n = 5). All data collection and analyses were adherent to Institutional Review Board approved protocols. Clinical characteristics, including age at diagnosis, race, histology, tumor size, tumor grade, and number of involved (+) axilla nodes were compared. Pair-wise comparison was done using two-tail t-test for age and tumor size, and Fisher’s exact test for race (Caucasian vs. African-American), histology, tumor grade (II vs. III) and number of (+) axilla nodes (none vs. one or more).

### Tissue dissociation and cell culture

After our surgical pathologists completed gross examination and inking of the tumor specimen, fresh tumor tissue was taken from the center of the tumor without interfering with margin assessment as determined by the pathologists. The tissues were stored in ice cold medium DMEM/F12 supplemented with 10% fetal bovine serum (FBS), penicillin and streptomycin. The fresh tumor tissue was kept on ice at 4°C until ready for processing within 6 hours from the excision time. If the tumor tissue weighed less than 0.5 gram (n = 5) (TB160 – TB165), the tissue was mechanically dissociated by mincing with scalpel and scissors to 1–2 mm^3^ in a 10 cm tissue culture plate. Fibroblast growth medium (DMEM supplemented with 10% FBS penicillin and streptomycin) was then added. After several days, outgrowth of spindle shaped cells was observed. Tissue debris and non-adherent cells were removed and medium changed between day 2–4. For tissues (n = 14) weighing more than 0.5 gram (TB71 - TB148) the tissue was minced as described above and then enzymatically dissociated in tissue digestion buffer containing collagenase I (Worthington), hyaluronidase (Sigma), Collagenase IV (Worthington) at 1 mg/ml of each enzyme in DMEM/F12 medium in a volume of 1:5 ratio of tumor to buffer (wt/vol) on a gyrating platform at 37^o^ C for 30 min. The digestion was quenched by addition of fibroblast growth medium and filtered through a 70 μm cell strainer. Cells were pelleted at 1500 rpm for 10 min. Tissue debris and non-adherent cells were removed during medium change between day 2 or 4. By 10 – 14 days, near confluent adherent spindle shaped cells were harvested using 0.25% trypsin in versene, washed and replated in fresh fibroblast growth medium. Medium was changed every 4 – 7 days. CAFs from early passages (passage 2–3) were harvested and the cell pellet was stored in RNA later (Applied Biosystems) at −80°C until RNA was isolated.

### RNA purification and microarrays

RNA purification was carried out using TRI Reagent® (Molecular Research Center) according to manufacturer’s recommendations. RNA quality was determined using the Bioanalyzer (Agilent). Only samples with RIN numbers > 7.5 were used for further studies. Equal amounts (400 ng) of total RNA was amplified as recommended by Illumina and hybridized to the HumanHT-12 v4 human whole genome bead arrays. Illumina BeadStudio v.3.0 software was used to export expression levels and detect p-values for each probe of each sample. Quality control of each array was performed using median Spearman correlation computed against all other arrays. Arrays whose median correlation differed from the global correlation by more than 8 absolute deviations were marked as outliers and not used for further analysis (resulting in the removal of one TNBC sample, TB147 (Table 1)). The remaining 19 arrays were then quantile-normalized between each other and filtered to remove non-informative probes (probes with a detection p-value > 0.05 in all samples). Between-batch normalization was performed using Distance Weighted Discrimination (DWD) approach [19] using 4 samples replicated in the 2 microarray batches. Average expression between replicates was used for data analysis. The data was submitted to GEO database ( http://www.ncbi.nlm.nih.gov/geo/) and available by using accession number GSE37614.

**Table 1:** List of samples used in gene expression analyses

**Subtype**	**Patient ID**	**b1**	**b2**	**set**
**TNBC**	TB123	x		training
	TB125	x		
	TB134	x	x	
	TB160		x	
	TB162		x	testing
	TB164		x	
	TB147		x	outlier
**ER+**	TB71	x		training
	TB75	x		
	TB130	x		
	TB163		x	
	TB165		x	
	TB98	x	x	testing
	TB120	x		
**Her2+**	TB76	x		training
	TB117	x	x	
	TB136	x		
	TB122	x	x	testing
	TB129		x	
**Her2+/ER+**	TB148		x	testing

List of samples divided into two batches (b1 and b2) including two samples from each subtype as an independent validation (testing) set as indicated.

### Flow cytometry analysis

1Adherent early passage CAFs were harvested with 0.05% trypsin/versene, washed in standard FACS buf-fer containing (5 ul/test) Fc blocking antibodies as recommended by the manufacture (Biolegend), and stained with the following directly conjugated antibodies for the evaluation of surface markers by flow cytometry analyses:

EpCAM: PE anti-human CD326 clone 9C4 (Biolegend) used at 1ug/ml; PE-F19: mouse anti-human FAP*α* monoclonal antibody (clone F19), used at 1/10 dilution, was purified from serum-free hybridoma supernatant as described [20,21]; CD45: APC mouse anti-human CD45 (BD Pharmingen) used at 20ul/test according to manufacturer's recommendation; CD31: APC anti-human CD31 clone WM59 (eBioscience) used at 5ug/ml.

### Independent validation

We randomly selected two samples from each Her2+, ER + and TNBC subtype as an independent validation set (testing set Table 1). One sample which was unique in its subtype classification in that the CAF was derived from a Her2 + and ER + breast cancer (TB148, Additional file 1: Table S1) was also added to the testing set in order to show how it would be classified based only on its gene expression profile. The training set used to select the genes that distinguish the 3 CAF subtypes included 3 Her2+, 5 ER + and 4 TNBC samples was analyzed with one way ANOVA to identify a list of significant genes with p-value < 0.05 used as a significance threshold. Expression patterns of the significant genes were used for Principal Component Analysis. Projection of training and testing set samples on the first two principal components was used to visualize relationship between samples.

### Differentially expressed genes

After the validation, a final list of significant genes differentially expressed between three classes of samples (Her2+, ER + and TNBC) was determined by using one way ANOVA on the full set of samples, except for the one Her2+/ER + sample (TB148). False discovery rate (FDR) was determined according to published protocol [22]. Significance for genes between each pair of groups was determined by Tukey post-hoc test. P-value <0.05 was set as a significance threshold.

### Gene enrichment analysis

Identification of biological functions and pathways overrepresented in any gene list was done using DAVID [23] and Ingenuity Pathway Analysis (IPA) software (Ingenuity Systems, Redwood City, CA). DAVID results were restricted to gene ontology (GO) terms, KEGG, and BIOCARTA pathways and Swiss-Prot keyword enrichments and filtered to satisfy FDR <5% and fold enrichment >2 criteria. Significance of IPA results was defined by Benjamini-Hochberg corrected for multiple testing p-value < 0.05.

### Heatmap

Heatmap was generated for a list of the 44 significant genes (with a fold change > 2) that distinguish Her2+ CAFs from both ER + and TNBC derived CAFs. Genes were hierarchically clustered using Spearman correlation distance and complete linkage. Heatmap color intensities were proportional to a value calculated as a ratio between the gene expression in a single sample and the geometric mean expression of the gene across all samples.

### qPCR validation

Expression of six genes, ITGA3, ITGA5, OXTR, WNT5B, BCAR1 and FZD1, as well as 3 endogenous controls (ec) RPL19, TBP and UBA5 were assessed by qRT-PCR in triplicates. Median Ct values for each gene were used for ΔΔCt analysis, where ΔCt was calculated against average Ct of the three endogenous controls and ΔΔCt calculated as difference between average ΔCt values of compared groups. Final fold change between a pair of groups was calculated as 2^ΔΔCt^. Significance of the difference between two groups was tested by two-tail t-test on ΔCt values. For comparison with expression values from microarrays, corrected for loading bias absolute expression values ***E*** for each gene ***G*** were calculated as follows: ***E*** = ***AE***_***G***_/(***AE***_***ec***_/avg(***AE***_***ec***_)), where absolute expression ***AE***_***G***_ = 2^40-***Ct***^, ***AE***_***ec***_ is an average ***AE*** between three endogenous controls and avg(***AE***_***ec***_) is an average of *AE*_*ec*_ taken across all samples. Expression values were then normalized for microarray and qRT-PCR data separately over three group average absolute expression values.

### Transwell migration assay

The migration properties of T47D (ATCC), a breast cancer cell line, known to have low migratory properties [24], was evaluated in the presence or absence of CAFs derived from ER, TNBC, and Her2+ breast cancer using a transwell assay. CAFs (1×10^4^ cells) from each of the three subtypes were seeded in 100 μl of DMEM containing 1% serum medium in the lower well of a Transwell chamber (Costar, Inc.) with 8 μm pore size polycarbonate filters and left to attach for 90mins. As control, medium containing no CAFs was placed in the lower well. T47D (1×10^4^ cells) were then seeded onto the upper chamber in 1% serum medium. Transwell chambers were incubated for 48 hours at 37°C and 5% CO_2_. Membranes were stained with DAPI (Invitrogen) for 15 min, rinsed with PBS and fixed with 10*%* buffered formalin (Fisher Scientific*,* SF100-20) for 15 min before imaging. The number of T47D cells that migrated onto the underside of the membrane was counted in 5 fields using a Nikon TE2000 inverted microscope at 10× magnification and plotted. Statistical evaluation was performed using Graph Pad Prism (GraphPad Software, Inc.)

## Results

### Isolation of CAFs from fresh human breast cancer samples

**Figure 2:**
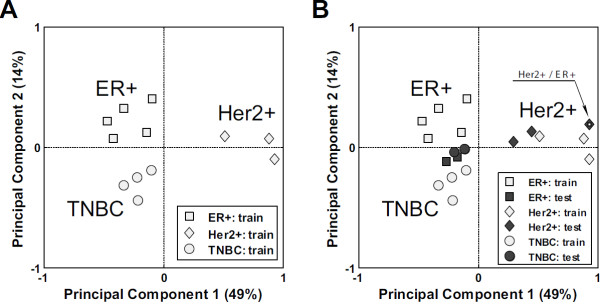
**CAFs derived from Her2+ breast cancer significantly enhances the migration of T47D cells *in vitro.*** In vitro transwell assays comparing T47D migration in the absence (orange) or presence of CAFs isolated from ER (black), Her2 (blue) and TNBC (green) primary human breast cancer tumors were performed. Each experiment was performed in duplicates using CAFs derived from at least two different patients. One CAF cell line of each subtype was tested in 2 independent experiments (open vs. closed circles). The second CAF cell line of each subtype (squares) was tested in duplicate in one independent experiment, for a total of 6 tests. Lines show mean ± SEM.

The clinical characteristics of the study cohort are summarized in Table 2. Detailed clinical characteristics of each tumor are provided in Additional file 1: Table S1. No significant differences were noted among the three subgroups, except for tumor grade (Table 2). The morphology of CAFs isolated from the 3 different breast cancer subtypes was similar (Figure 1). Further phenotypic characterization using flow cytometry analysis demonstrated that >95% of these cells expressed fibroblast activation protein (FAP), a previously identified marker of cancer associated fibroblasts [25-28]. Moreover, >99% of the cells were negative for the epithelial cell adhesion molecule (EpCAM), a breast cancer epithelial cell surface marker [12]; CD31, also known as platelet endothelial cell adhesion molecule (PECAM-1), an endothelial cell marker, and CD45, a pan-leukocyte marker (Figure 2, lower panel). Moreover, these CAFs uniformly expressed vimentin and collagen by immunohistochemistry (data not shown).

**Table 2:** Clinical characteristics of breast cancer study cohort

		**Overall**	**TNBC**	**ER+**	**Her2+**	**p-values**		
						**TNBC vs. ER+**	**TNBC vs. Her2+**	**ER+ vs. Her2+**
**n**		**20**	**7**	**7**	**6**			
**Age at diagnosis** mean ± standard deviation		52 ± 16	47 ± 14	59 ± 18	49 ± 16	0.21	0.83	0.33
**Ethnicity**								
	Caucasian	10	3	5	2	0.59	1	0.56
	African American	9	4	2	3			
	Asian	1	0	0	1			
**Invasive carcinoma histology**								
	ductal	14	7	3	6	0.07	1	0.19
	lobular	6	0	4	0			
**Tumor size (cm)** mean ± standard deviation		4.8 ± 4.2	3.0 ± 1.1	4.9 ± 2.5	5.7 ± 7.4	0.06	0.35	0.81
T1	<2 cm	4	1	1	2			
T2	2.1 - 5 cm	10	6	2	2			
T3	>5 cm	6	0	4	2			
**Tumor grade**								
	I	0	0	0	0			
	II	3	1	3	0	0.03	1	0.03
	III	11	6	0	4			
	not assessed	6	0	4	2			
**No. of involved axilla node(s)** mean ± standard deviation		5.5 ± 7.8	4.1 ± 8.6	6.4 ± 8.4	6.0 ± 7.0	0.10	0.56	0.52
	0	8	5	1	2			
	1-3	4	0	4	0			
	4-9	3	1	0	1			
	>9	4	1	2	2			
	not assessed	1	0	0	1			
**Receptor status**								
	ER+	8	0	7	1			
	PR+	7	0	7	0			
	Her2+	6	0	0	6			

### Gene expression profile analyses of CAFs derived from TNBC, ER + and Her2+ breast cancer

RNA isolated from the early passage CAFs were assayed for gene expression and randomly assigned to two sample sets, namely, training and testing sets (Table 1) to perform independent validation. Using one-way ANOVA on the training set (4 TNBC samples, 5 ER + samples and 3 Her2+ samples)), we identified 782 genes that were differentially expressed between TNBC, ER + and Her2+ samples (p-value < 0.05). In order to visualize the relationships between the sample types, we performed unsupervised Principal Component Analysis using the 782 significant genes (Figure 2A). This type of plot reflects the similarities and differences between all samples in relation to the 782 significant genes. It should be noted that the first principal component plotted on the X axis accounts for 49% of the variation in the data and indicates that there are significant differences between the CAFs derived from the Her2+ cancers and both the TNBC and ER + breast cancers, as these samples are equally separated from the Her2+ samples along the X axis. The second principal component plotted on the Y axis accounts for only 14% of the gene expression variation between all samples. It captures putative differences between the ER + and TNBC samples and indicates that the expression profiles are much more similar between these two subtypes.

**Figure 3:**
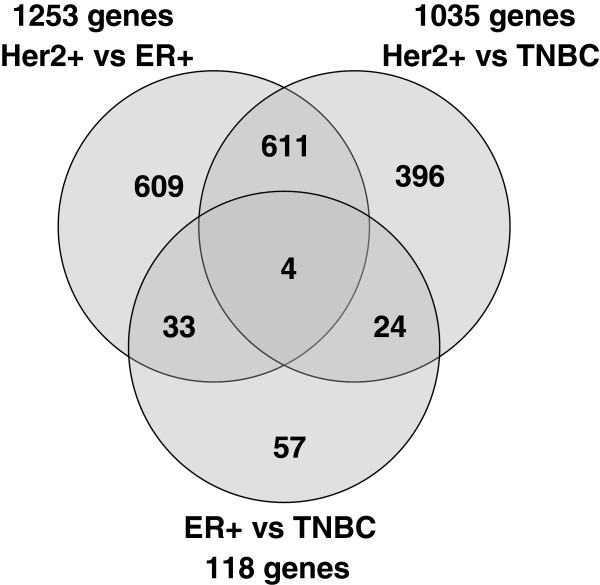
**Characterization of CAFs from breast cancer subtypes by morphology (light microscopy) and flow cytometry analysis.** Top panel, 20x magnification light microscopy pictographs of **a**) ER+; **b**) TNBC and **c**) Her2+ breast cancer derived CAFs; Lower panel, histograms (dark solid line) depicting CAFs staining for (left to right): EpCAM, FAP, CD45, and CD31; light grey lines depict histogram of CAFs staining with isotype control antibodies.

We then determined whether the training set principal components could also distinguish the new Her2+, ER + and TNBC patient samples thus validating our initial observations. Figure 2A shows the separation of the 12 samples representing the 3 original sample types in the training set that we used to select the significant genes that defined this separation. Figure 2 Bconfirms these genes also identify the subtype differences in new samples analyzed as an independent validation set and included two new Her2+ samples and t two new ER + and two new TNBC samples. The new Her2+ samples clearly cluster with the Her2+ samples in the training set while the new ER + and TNBC samples once again cluster with the ER + and TNBC training set samples. Although the ER + and TNBC derived CAFs appear to self segregate along the 2^nd^ principal component in the training set (Figure 2A), no significant differences in gene expression were detected between the ER + and TNBC CAFs in the testing set (Figure 2B). This indicates that there is a high degree of gene expression similarity in the CAFs associated with the ER + and TNBC cancer subtypes.

It should also be noted that new sample TB148, which is both Her2+ and ER+, co-segregates with the Her2+ samples which were all ER- (Figure 2B), indicating the presence of a gene expression profile more similar to the Her2+ CAFs and not the ER + CAF sample group. This indicates a dominance of Her2+ CAF gene expression signature over ER + CAF signature.

We also combined the expression data for all samples (except for the Her2+/ER + TB148) to take advantage of the larger sample size and ran one way ANOVA to define a final list of significant genes differentially expressed between Her2+, ER + and TNBC in the larger data set. We found 1829 differentially expressed genes with p-value < 0.05 and estimated false discovery rate of 28%. When the relationships between the different CAF subtypes were reassessed using Principal Component Analysis with the new gene set, we found the same cancer subtype specific differences as demonstrated on training subset (Figure 2A).

**Figure 4:**
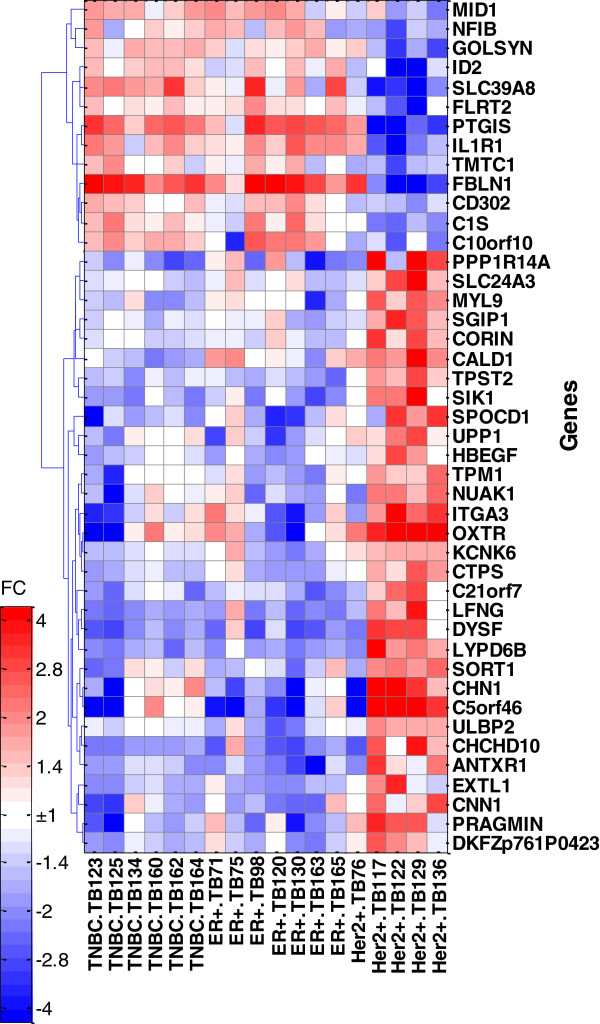
**Relationship between Her2+, ER + and TNBC classes of samples visualized by Principal Component Analysis (PCA) on the training set samples using expression of genes differentially expressed between the three classes. A**. Training set samples **B**. Projection of testing set samples on the first and second principal components derived from the training set. White square in dark grey diamond indicates tested sample with double diagnosis Her2+/ER +.

The number of significant genes identified by pair-wise comparisons (Tukey post-hoc test) between the three classes of patient samples, i.e. Her2+ vs ER+, Her2+ vs. TNBC and ER + vs TNBC samples, are presented in the Venn diagram in Figure 3. These results quantify the visual interpretation of Principal Component Analysis demonstrating that while 1,800 genes were significantly differentially expressed between Her2+ and either ER + or TNBC, only 118 genes were significantly different between ER + and TNBC derived CAFs. Further studies with increased number of samples for ER + and TNBC derived CAFs will be required to identify genes that can discrimi-nate those 2 classes, if they exist. A gene expression heat map for the 44 most changed unique genes (fold change > 2) which were common to the Her2+ vs ER + and Her2 + vs TNBC comparisons are shown in Figure 4.

**Figure 5:**
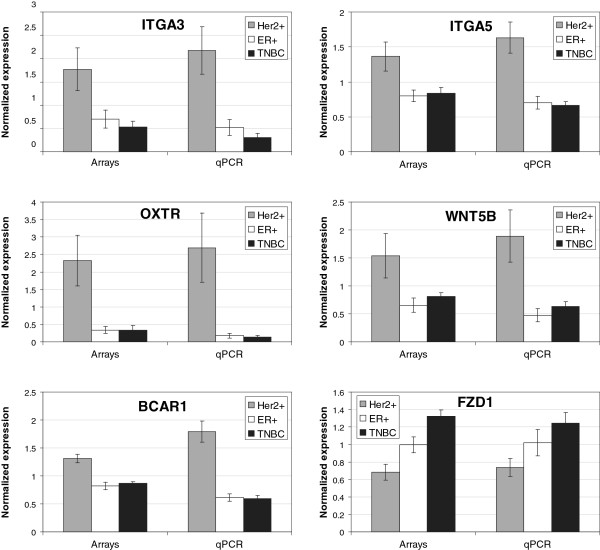
Venn diagram for genes common between three pair-wise comparisons of Her2+, ER + and TNBC classes of samples.

### Functions and pathways over-represented in the list of genes that distinguish Her2+ from ER + and TNBC CAFs

We compared the two significant gene lists for Her2+ vs ER + and Her2+ vs TNBC to identify functions or pathways that might be over-represented among the differentially expressed genes. Results with DAVID software analyses [23] are shown in Additional file 2: Table S2 for the Her2+ vs ER + 1253 significant genes, and in Additional file 3: Table S3 for Her2+ vs TNBC 1035 significant genes. Enrichment of nine functional categories associated with cytoskeleton and extracellular matrix were found to be significant in both comparisons.

Ingenuity pathway analysis was done for a list of 615 genes common between Her2+ vs ER + and Her2+ vs. TNBC comparisons. A list of significantly enriched canonical pathways is presented in Table 3. Pathways involving extracellular matrix/integrin signaling were found to be significantly up-regulated in CAFs derived from Her2+ cancer, further supporting the DAVID results. It should be noted that 92% (61 of the 66 unique) of the genes associated with the ingenuity pathways are upregulated in Her2+ supporting the hypothesis that those pathways are more active in CAFs derived from Her2+ breast cancer as compared to those derived from the ER + and TNBC breast cancers.

**Table 3:** Canonical pathways upregulated in Her2+ compared to ER + and TNBC samples

**Enriched ingenuity canonical pathways**	***pval***	**# of genes**				**Genes**
		**P**	**L**	**↑**	**↓**	
Actin Cytoskeleton Signaling	0.0002	226	20	20	0	PFN1↑, MYL6↑, CFL1↑, ARPC5L↑, CSK↑, HRAS↑, ITGA5↑, IQGAP1↑, ITGA3↑, BCAR1↑, ACTG1↑, MYL9↑, MYL12A↑, PIP5K1C↑, ARPC2↑, RHOA↑, MYH9↑, VCL↑, ACTN1↑, MSN↑
Integrin Signaling	0.0008	205	18	18	0	MAP3K11↑, RHOC↑, ARPC5L↑, ILK↑, HRAS↑, PLCG1↑, ITGA5↑, TNK2↑, ITGA3↑, BCAR1↑, ACTG1↑, NCK2↑, ARF1↑, MYL12A↑, ARPC2↑, RHOA↑, VCL↑, ACTN1↑
Regulation of Actin-based Motility by Rho	0.001	87	11	11	0	MYL9↑, MYL12A↑, PFN1↑, CFL1↑, MYL6↑, ARPC5L↑, PIP5K1C↑, RHOC↑, ARPC2↑, RHOA↑, ARHGDIA↑
Rac Signaling	0.002	117	12	12	0	RELA↑, MAP3K11↑, CFL1↑, ARPC5L↑, PIP5K1C↑, ARPC2↑, RHOA↑, ITGA5↑, HRAS↑, SH3RF1↑, ITGA3↑, IQGAP1↑
Cdc42 Signaling	0.003	142	13	13	0	MPRIP↑, MAP3K11↑, CFL1↑, MYL6↑, ARPC5L↑, ITGA5↑, TNK2↑, ITGA3↑, IQGAP1↑, HLA-F↑, MYL9↑, MYL12A↑, ARPC2↑
ILK Signaling	0.005	182	15	14	1	RELA↑, CFL1↑, MYL6↑, RHOC↑, ILK↑, ACTG1↑, MYC↓, NCK2↑, MYL9↑, TGFB1I1↑, PPP2R1A↑, FLNA↑, RHOA↑, MYH9↑, ACTN1↑
RhoA Signaling	0.006	107	11	11	0	MYL9↑, MYL12A↑, PFN1↑, CFL1↑, MYL6↑, ARPC5L↑, PIP5K1C↑, ARPC2↑, RHOA↑, ACTG1↑, MSN↑
PI3K/AKT Signaling	0.010	129	11	10	1	RELA↑, PPP2R1A↑, NFKBIA↓, YWHAH↑, TSC2↑, TYK2↑, ILK↑, ITGA5↑, HRAS↑, ITGA3↑, NFKBIB↑
Germ Cell-Sertoli Cell Junction Signaling	0.010	159	13	13	0	MAP3K11↑, RHOC↑, TUBB2A↑, ILK↑, HRAS↑, ITGA3↑, IQGAP1↑, BCAR1↑, ACTG1↑, TUBB6↑, SORBS1↑, RHOA↑, ACTN1↑
Cardiac Hypertrophy Signaling	0.010	228	16	14	1	MAP3K11↑, CALM1↑, MYL6↑, RHOC↑, PLCG1↑, HRAS↑, PPP3CC↑, EIF2B2↑, MYL9↑, GNB1↑, PLCD3↓, MYL12A↑, PLCB4↑, RHOA↑, MAPKAPK2↑, HSPB1↑
Phospholipase C Signaling	0.01	243	16	14	0	RELA↑, MYL6↑, CALM1↑, RHOC↑, PLCG1↑, ITGA5↑, PPP1R14A↑, HRAS↑, ARHGEF17↑, PPP3CC↑, ITGA3↑, MYL9↑, GNB1↑, PLCB4↑, MYL12A↑, RHOA↑
Protein Kinase A Signaling	0.01	306	19	13	3	RELA↑, YWHAH↑, MYL6↑, CALM1↑, PPP1R14A↑, PLCG1↑, PPP1R11↑, PPP3CC↑, MYL9↑, GNB1↑, PLCD3↓, MYL12A↑, PLCB4↑, NFKBIA↓, PDE7B↓, FLNA↑, RHOA↑, NFKBIB↑, PDE6D↑
FAK Signaling	0.01	98	9	9	0	CSK↑, PLCG1↑, ITGA5↑, HRAS↑, VCL↑, ITGA3↑, TNS1↑, BCAR1↑, ACTG1↑
fMLP Signaling in Neutrophils	0.01	117	10	6	0	GNB1↑, RELA↑, PLCB4↑, NFKBIA↓, CALM1↑, ARPC5L↑, ARPC2↑, HRAS↑, PPP3CC↑, NFKBIB↑
Axonal Guidance Signaling	0.04	422	21	21	0	KLC1↑, PFN1↑, GLI2↑, PLXNA3↑, MYL6↑, CFL1↑, ARPC5L↑, TUBB2A↑, HRAS↑, TGA5↑, PPP3CC↑, ITGA3↑, BCAR1↑, NCK2↑, MYL9↑, GNB1↑, PLCB4↑, MYL12A↑, TUBB6↑, ARPC2↑, RHOA↑
Neuregulin Signaling	0.04	95	8	6	2	MYC↓, PICK1↑, PLCG1↑, ITGA5↑, HBEGF↑, HRAS↑, ITGA3↑, STAT5B↓
PAK Signaling	0.05	104	8	8	0	NCK2↑, MYL9↑, MYL12A↑, CFL1↑, MYL6↑, ITGA5↑, HRAS↑, ITGA3↑
Virus Entry via Endocytic Pathways	0.05	92	8	8	0	AP2M1↑, FLNA↑, PLCG1↑, ITGA5↑, HRAS↑, ITGA3↑, ACTG1↑, DNM2↑

*pval* = Benjamini-Hochberg corrected p-value, P = total number of genes known to be involved in the pathway, L = number of genes from the pathway that were also in the list of significant genes. ↑ = number of genes significantly upregulated in Her2+, ↓ = number of genes significantly downregulated in Her2+. The 18 significantly enriched pathways share 66 unique genes with 61 of those upregulated in Her2+ compared to ER + and TNBC.

### Q-RT-PCR validation of individual gene expression data in CAFs

To confirm differential gene expression levels in the three breast cancer subtypes, Her2+, ER + and TNBC, we selected 6 genes (*ITGA3, ITGA5, OXTR, WNT5B, BCAR1, FZD1)* with significantly different levels of expression based on our microarray studies and validated their expression levels by qRT-PCR. Fold changes in expression based on the arrays ranged from 1.5 fold to 6.9 fold. Five of the 6 genes that were found to be expressed at higher levels in the Her2+ samples were also significantly different in the Her2+/ER + qRT-PCR comparison; and 4 of those 5 genes that were significantly different in the Her2+/TNBC array comparison were also significantly different by qRT-PCR comparison (Figure 5. and Additional file 4: Table S4). Expression ratios by qRT-PCR were highly consistent with array values and overall somewhat higher by qRT-PCR as expected. One gene, FZD1, which was expressed at lower levels in CAFs derived from Her2+ breast cancer by array analyses, was also significantly lower by qRT-PCR in the Her2/TNBC comparison but was not significantly different in the ER/TNBC comparison (*P* = 0.2) although fold change values were similar by qRT-PCR (TNBC/ER + = 1.33 for microarrays and 1.39 for qPCR).

**Figure 6:**
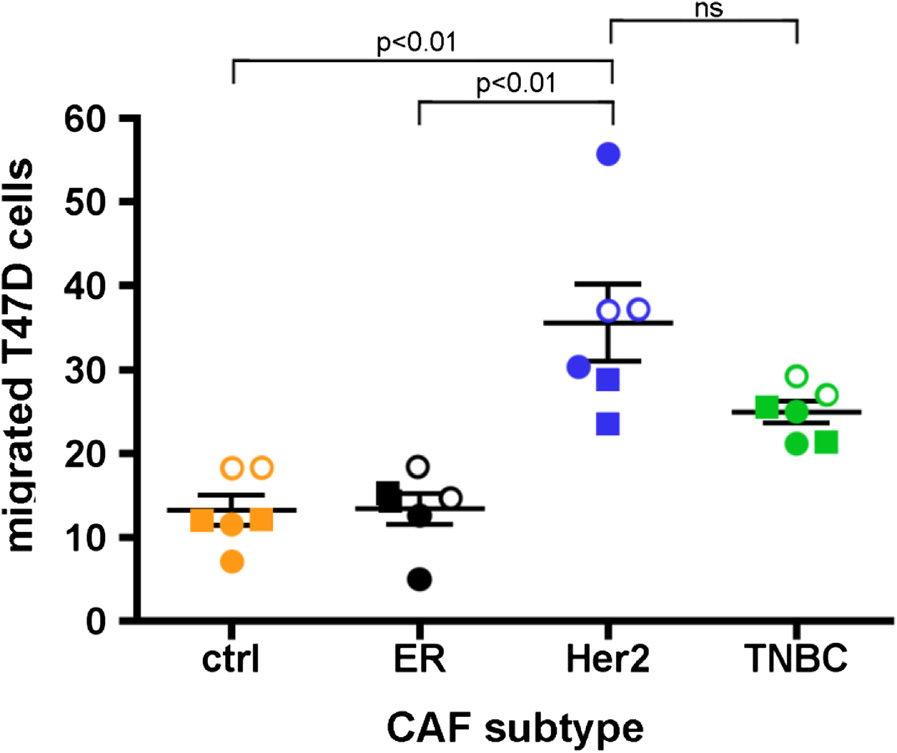
**Heat map of expression for 44 genes with the greatest differences between Her2+ vs. ER + and Her2+ vs. TNBC comparisons.** FC = fold change from geometrical mean of expression across all samples.

### Her2 CAFs enhanced the migratory phenotype of breast cancer cells *in vitro*

To explore whether CAFs derived from various breast cancer subtypes can differentially enhance the migratory phenotype of breast cancer cells, we performed in vitro transwell assays comparing the migration of breast cancer cells cultured in the presence or absence of CAFs isolated from ER+, Her2+ and TNBC. The number of migrated T47 cells onto the membrane surface that was facing the lower chamber was counted. Results were analyzed by unpaired Kruskal-Wallis test. The level of statistical significance was taken as P < 0.05. As our gene expression profile results have predicted, CAFs derived from Her2+ breast cancer significantly enhanced the migration of T47D (Figure 6).

## Discussion

Robust evidence is now available that underscores the role of CAFs in tumor progression [8,28-33]. Previous gene expression profile analyses comparing CAFs and fibroblasts derived from matched normal adjacent breast tissues have demonstrated significant differences between the CAF and their normal counterparts but, to the best of our knowledge, no prior studies have addressed whether CAFs derived from various breast cancer subtypes harbor subtype specific gene expression signatures. In this study we demonstrate for the first time that CAFs from several breast cancer subtypes exhibit subtype-specific gene expression profiles. Specifically, we show that the gene expression profile of CAFs derived from Her2+ breast cancers are significantly different from CAFs derived from ER + or TNBC breast cancers.

Heterogeneity among fibroblasts has been described in various organ sites including lung, skin, sclera and orbit [34]. Furthermore, Sugimoto and coworkers demonstrated that the expression of various fibroblast markers are heterogeneous within the tumor stroma in mouse breast and pancreatic tumor models using immunohistochemical analyses [35]. Several studies have generated gene expression profiles from breast cancer-associated fibroblasts but none of these studies have stratified their results based on tumor subtypes. Work by Allinen and coworkers evaluated gene expression profiles of breast cancer stromal cells which were isolated by negatively selecting out epithelial cells, lymphocytes and endothelial cells [12]. Work described by Singer et al. compared gene expression profiles of stromal fibroblasts derived from 10 invasive breast cancers with stromal fibroblasts derived from normal breast tissues of 10 women undergoing breast reduction surgery [16]. Their results demonstrated increased expression of tumor promotion-associated genes in the pooled CAFs. Work by Bauer et al. (2010) evaluated gene expression profiles of fibroblasts derived from 6 matched breast cancers and adjacent normal breast tissues [13] and found distinct differences between CAFs and normal fibroblasts, specifically in genes related to paracrine or intracellular signaling, transcriptional regulation, extracellular matrix and cell adhesion/migration. However, all of the above studies were not designed to test subtype specific differences in CAFs due to these studies’ relatively small sample size. In addition, when tumor subtype data were reported, the less common breast cancer subtypes, i.e., Her2+ or TNBC cancer, were underrepresented.

Our results showed that CAFs derived from Her2+ breast cancers significantly up-regulated pathways associated with actin cytoskeleton and integrin signaling (Table 3). Integrins mediate cell attachment with extracellular matrix (ECM) to provide traction necessary for cell motility and invasion. These upregulated signaling pathways may have contributed to the elevated migratory phenotype of breast cancer cells (T47D) in our *in vitro* transwell assays (Figure 1).

The extracellular matrix and integrins collaborate to regulate gene expression associated with cell growth, differentiation and survival; all of which are deregulated during cancer progression and metastasis. A recent study using a three-dimensional squamous cell carcinoma (SCC)/fibroblast co-culture model elegantly demonstrated the role of three genes, integrin α3, integrin α5 and Rho, in promoting a fibroblast-led collective invasion of SCC cells into the extracellular matrix [17]. Interestingly, all three genes were significantly up-regulated in CAFs derived from Her2+ breast cancer with integrin signaling as the second most enriched pathway (Table 3). Moreover, many of the genes and pathways downstream of integrin signaling are also significantly upregulated in Her2+ CAFs. These include focal adhesion kinase (FAK), Rac and Rho signaling pathways as well as several members of the mitogen-activated protein kinases (MAPKs), further underscoring the importance of integrin signaling in CAF. In addition to the well-established role of integrins in migration and invasion, integrins can also regulate cell proliferation, including mammary gland proliferation [36] through integrin-linked kinase (ILK) [37], which was also noted to be significantly upregulated in HER2+ derived CAFs. These characteristic differences in CAFs derived from Her2+ breast cancer may contribute to the aggressiveness of this particular breast cancer subtype which is known to have an increased propensity for local and distant recurrence [3]. In addition, the sites of distant metastasis appear to differ according to breast cancer subtype with Her2+ breast cancer having a higher rate of brain, liver, and lung metastases than ER + breast cancer [38]. The role of CAF in contributing to a subtype-specific trophism for the various distant metastatic sites is unknown.

Gene expression profile differences between CAFs derived from ER + and TNBC breast cancer were less pronounced and we were unable to confirm them with independent validation set using the limited sample numbers (Figure 2B). While it is possible that true differences may exist among these two subtypes, a larger number of samples would be required to find those differences with an acceptable false discovery rate.

## Conclusions

Our results show that subtype specific changes exist in CAFs derived from breast cancer. In the case of Her2+ breast cancer, a more aggressive breast cancer subtype with known increased risk of local and distant recurrence, CAFs may augment the invasive properties of the tumor cells via pathways associated with cytoskeleton and integrin signaling. Our findings also provided molecular evidence supporting a recently proposed tumor-stroma co-evolution hypothesis which suggested that the tumor microenvironment, e.g. CAFs, may adopt specific changes to optimize the survival/propagation of a specific tumor cell type [5]. Whether these programmatic differences in CAFs result from epigenetic changes or whether these differences are due to heterogeneity within the CAF population, i.e. proportion of resident fibroblasts vs. recruited fibroblasts, or fibroblasts derived from epithelial mesenchymal transition are unknown. In addition, whether CAFs contribute to tumor progression in a subtype specific manner is unknown. How CAFs and other components of the tumor microenvironment drive or are being driven by the tumor cells to promote the propagation and maintenance of a specific tumor subtype will be the subject of future work.

## Competing interests

The authors declare no conflict of interest.

## Authors' contributions

JT, AVK and LS designed the study; JT, AVK, LC, CS performed the experiments described in this study; JT, AVK, LC, MH, LS and EP contributed to the writing of the manuscript. All authors read and approved the final manuscript.

## Pre-publication history

The pre-publication history for this paper can be accessed here:

http://www.biomedcentral.com/1755-8794/5/39/prepub

## Supplementary Material

Additional file 1 **Table S1.**Clinical Characteristics of Study Cohort. Click here for file

Additional file 2 **Table S2.**Annotation categories enriched in the list of genes significantly differentially expressed in Her2+ compared to ER+ samples as determined by DAVID software. Cat=category, Term=enriched annotation term, Enr=enrichment, TN=enrichment of the Term in Her2+ vs. TNBC comparison, Sens=sensitivity in a form K/N(P%), where K=number of genes in the list, N=total known number of genes, P=K/N in percentage. P=Fisher exact p-value for enrichment, FDR=false discovery rate, ↑ = number of genes upregulated in Her2+, ↓ = number of genes downregulated in Her2+, SP.KW = SwissProt keyword, KEGG=KEGG pathway, GO=gene ontology, BP=biological process, FM=molecular function, CC=cellular component. Click here for file

Additional file 3 **Table S3.**Annotation categories enriched in the list of genes significantly differentially expressed in Her2+ compared to TNBC samples as determined by DAVID software. Cat=category, Term=enriched annotation term, Enr=enrichment, ER+=enrichment of the Term in Her2+ vs. ER+ comparison, Sens=sensitivity in a form K/N(P%), where K=number of genes in the list, N=total known number of genes, P=K/N in percentage. P=Fisher exact p-value for enrichment, FDR=false discovery rate, ↑ = number of genes upregulated in Her2+, ↓ = number of genes downregulated in Her2+, SP.KW = SwissProt keyword, GO=gene ontology, BP=biological process, FM=molecular function, CC=cellular component. Click here for file

Additional file 4 **Table S4.**Fold changes and p-values obtained by qRT-PCR validation experiment for 6 genes found to be significantly different in either Her2+ vs ER+, Her2+ vs TNBC or ER+ vs TNBC comparison in microarrays data. FC=fold change, P=significance by t-test. Visual comparison of expression values between microarrays and qRT-PCR are presented in Figure 6. Click here for file
