# Supplementary material for: Human breast cancer associated fibroblasts exhibit subtype specific gene expression profiles
Source: BMC Med Genomics. 2012 Sep 6;5:39. doi: 10.1186/1755-8794-5-39 (PMC3505468; doi:10.1186/1755-8794-5-39)
Supplement: Additional file 1 — Table S1.Clinical Characteristics of Study Cohort. [file 1755-8794-5-39-S1.docx]

| **Table 1. Clinical characteristics of breast cancer study cohort** | | | | |  |  |  |  |
| --- | --- | --- | --- | --- | --- | --- | --- | --- |
|  |  | **Overall** | **TNBC** | **ER+** | **Her2+** | **p-values** | | |
|  |  |  |  |  |  | **TNBC vs. ER+** | **TNBC vs. Her2+** | **ER+ vs. Her2+** |
| **n** |  | 20 | 7 | 7 | 6 |  |  |  |
|  |  |  |  |  |  |  |  |  |
| **Age at diagnosis**  mean ± standard deviation | | 52 ± 16 | 47 ± 14 | 59 ± 18 | 49 ± 16 | 0.21 | 0.83 | 0.33 |
|  |  |  |  |  |  |  |  |  |
| **Ethnicity** |  |  |  |  |  |  |  |  |
|  | Caucasian | 10 | 3 | 5 | 2 | 0.59 | 1 | 0.56 |
|  | African American | 9 | 4 | 2 | 3 |  |  |  |
|  | Asian | 1 | 0 | 0 | 1 |  |  |  |
|  | |  |  |  |  |  |  |  |
| **Invasive carcinoma histology** | |  |  |  |  |  |  |  |
|  | ductal | 14 | 7 | 3 | 6 | 0.07 | 1 | 0.19 |
|  | lobular | 6 | 0 | 4 | 0 |  |  |  |
|  |  |  |  |  |  |  |  |  |
| **Tumor size (cm)**  mean ± standard deviation | | 4.8 ± 4.2 | 3.0 ± 1.1 | 4.9 ± 2.5 | 5.7 ± 7.4 | 0.06 | 0.35 | 0.81 |
| T1 | < 2 cm | 4 | 1 | 1 | 2 |  |  |  |
| T2 | 2.1 - 5 cm | 10 | 6 | 2 | 2 |  |  |  |
| T3 | > 5 cm | 6 | 0 | 4 | 2 |  |  |  |
|  |  |  |  |  |  |  |  |  |
| **Tumor grade** |  |  |  |  |  |  |  |  |
|  | I | 0 | 0 | 0 | 0 |  |  |  |
|  | II | 3 | 1 | 3 | 0 | 0.03 | 1 | 0.03 |
|  | III | 11 | 6 | 0 | 4 |  |  |  |
|  | not assessed | 6 | 0 | 4 | 2 |  |  |  |
|  |  |  |  |  |  |  |  |  |
| **No. of involved axilla node(s)**  mean ± standard deviation | | 5.5 ± 7.8 | 4.1 ± 8.6 | 6.4 ± 8.4 | 6.0 ± 7.0 | 0.10 | 0.56 | 0.52 |
|  | 0 | 8 | 5 | 1 | 2 |  |  |  |
|  | 1-3 | 4 | 0 | 4 | 0 |  |  |  |
|  | 4-9 | 3 | 1 | 0 | 1 |  |  |  |
|  | >9 | 4 | 1 | 2 | 2 |  |  |  |
|  | not assessed | 1 | 0 | 0 | 1 |  |  |  |
|  | |  |  |  |  |  |  |  |
| **Receptor status** | |  |  |  |  |  |  |  |
|  | ER+ | 8 | 0 | 7 | 1 |  |  |  |
|  | PR+ | 7 | 0 | 7 | 0 |  |  |  |
|  | Her2+ | 6 | 0 | 0 | 6 |  |  |  |
