# Supplementary material for: Human breast cancer associated fibroblasts exhibit subtype specific gene expression profiles
Source: BMC Med Genomics. 2012 Sep 6;5:39. doi: 10.1186/1755-8794-5-39 (PMC3505468; doi:10.1186/1755-8794-5-39)
Supplement: Additional file 2 — Table S2.Annotation categories enriched in the list of genes significantly differentially expressed in Her2+ compared to ER+ samples as determined by DAVID software. Cat=category, Term=enriched annotation term, Enr=enrichment, TN=enrichment of the Term in Her2+ vs. TNBC comparison, Sens=sensitivity in a form K/N(P%), where K=number of genes in the list, N=total known number of genes, P=K/N in percentage. P=Fisher exact p-value for enrichment, FDR=false discovery rate, ↑ = number of genes upregulated in Her2+, ↓ = number of genes downregulated in Her2+, SP.KW = SwissProt keyword, KEGG=KEGG pathway, GO=gene ontology, BP=biological process, FM=molecular function, CC=cellular component. [file 1755-8794-5-39-S2.docx]

**Table 2. List of samples divided into two batches (b1 and b2) including two samples from each subtype as an independent validation (testing) set as indicated.**

| **Subtype** | **Patient ID** |  | **b1** | **b2** | **set** |
| --- | --- | --- | --- | --- | --- |
| **TNBC** | TB123 |  | x |  | training |
|  | TB125 |  | x |  |  |
|  | TB134 |  | x | x |  |
|  | TB160 |  |  | x |  |
|  | TB162 |  |  | x | testing |
|  | TB164 |  |  | x |  |
|  | TB147 |  |  | x | outlier |
| **ER+** | TB71 |  | x |  | training |
|  | TB75 |  | x |  |  |
|  | TB130 |  | x |  |  |
|  | TB163 |  |  | x |  |
|  | TB165 |  |  | x |  |
|  | TB98 |  | x | x | testing |
|  | TB120 |  | x |  |  |
| **Her2+** | TB76 |  | x |  | training |
|  | TB117 |  | x | x |  |
|  | TB136 |  | x |  |  |
|  | TB122 |  | x | x | testing |
|  | TB129 |  |  | x |  |
| **Her2+/ER+** | TB148 |  |  | x | testing |
