# Supplementary material for: Human breast cancer associated fibroblasts exhibit subtype specific gene expression profiles
Source: BMC Med Genomics. 2012 Sep 6;5:39. doi: 10.1186/1755-8794-5-39 (PMC3505468; doi:10.1186/1755-8794-5-39)
Supplement: Additional file 3 — Table S3.Annotation categories enriched in the list of genes significantly differentially expressed in Her2+ compared to TNBC samples as determined by DAVID software. Cat=category, Term=enriched annotation term, Enr=enrichment, ER+=enrichment of the Term in Her2+ vs. ER+ comparison, Sens=sensitivity in a form K/N(P%), where K=number of genes in the list, N=total known number of genes, P=K/N in percentage. P=Fisher exact p-value for enrichment, FDR=false discovery rate, ↑ = number of genes upregulated in Her2+, ↓ = number of genes downregulated in Her2+, SP.KW = SwissProt keyword, GO=gene ontology, BP=biological process, FM=molecular function, CC=cellular component. [file 1755-8794-5-39-S3.docx]

**Table 3. Canonical pathways upregulated in Her2+ compared to ER+ and TNBC samples.** *pval*=Benjamini-Hochberg corrected p-value, P=total number of genes known to be involved in the pathway, L=number of genes from the pathway that were also in the list of significant genes. ↑ = number of genes significantly upregulated in Her2+, ↓ = number of genes significantly downregulated in Her2+. The 18 significantly enriched pathways share 66 unique genes with 61 of those upregulated in Her2+ compared to ER+ and TNBC.

| **Enriched Ingenuity**  **Canonical Pathways** | ***pval*** | **# of genes** | | | | **Genes** |
| --- | --- | --- | --- | --- | --- | --- |
|  |  | **P** | **L** | **↑** | **↓** |  |
| Actin Cytoskeleton Signaling | 0.0002 | 226 | 20 | 20 | 0 | PFN1↑, MYL6↑, CFL1↑, ARPC5L↑, CSK↑, HRAS↑, ITGA5↑, IQGAP1↑, ITGA3↑, BCAR1↑, ACTG1↑, MYL9↑, MYL12A↑, PIP5K1C↑, ARPC2↑, RHOA↑, MYH9↑, VCL↑, ACTN1↑, MSN↑ |
| Integrin Signaling | 0.0008 | 205 | 18 | 18 | 0 | MAP3K11↑, RHOC↑, ARPC5L↑, ILK↑, HRAS↑, PLCG1↑, ITGA5↑, TNK2↑, ITGA3↑, BCAR1↑, ACTG1↑, NCK2↑, ARF1↑, MYL12A↑, ARPC2↑, RHOA↑, VCL↑, ACTN1↑ |
| Regulation of Actin-based Motility by Rho | 0.001 | 87 | 11 | 11 | 0 | MYL9↑, MYL12A↑, PFN1↑, CFL1↑, MYL6↑, ARPC5L↑, PIP5K1C↑, RHOC↑, ARPC2↑, RHOA↑, ARHGDIA↑ |
| Rac Signaling | 0.002 | 117 | 12 | 12 | 0 | RELA↑, MAP3K11↑, CFL1↑, ARPC5L↑, PIP5K1C↑, ARPC2↑, RHOA↑, ITGA5↑, HRAS↑, SH3RF1↑, ITGA3↑, IQGAP1↑ |
| Cdc42 Signaling | 0.003 | 142 | 13 | 13 | 0 | MPRIP↑, MAP3K11↑, CFL1↑, MYL6↑, ARPC5L↑, ITGA5↑, TNK2↑, ITGA3↑, IQGAP1↑, HLA-F↑, MYL9↑, MYL12A↑, ARPC2↑ |
| ILK Signaling | 0.005 | 182 | 15 | 14 | 1 | RELA↑, CFL1↑, MYL6↑, RHOC↑, ILK↑, ACTG1↑, MYC↓, NCK2↑, MYL9↑, TGFB1I1↑, PPP2R1A↑, FLNA↑, RHOA↑, MYH9↑, ACTN1↑ |
| RhoA Signaling | 0.006 | 107 | 11 | 11 | 0 | MYL9↑, MYL12A↑, PFN1↑, CFL1↑, MYL6↑, ARPC5L↑, PIP5K1C↑, ARPC2↑, RHOA↑, ACTG1↑, MSN↑ |
| PI3K/AKT Signaling | 0.010 | 129 | 11 | 10 | 1 | RELA↑, PPP2R1A↑, NFKBIA↓, YWHAH↑, TSC2↑, TYK2↑, ILK↑, ITGA5↑, HRAS↑, ITGA3↑, NFKBIB↑ |
| Germ Cell-Sertoli Cell Junction Signaling | 0.010 | 159 | 13 | 13 | 0 | MAP3K11↑, RHOC↑, TUBB2A↑, ILK↑, HRAS↑, ITGA3↑, IQGAP1↑, BCAR1↑, ACTG1↑, TUBB6↑, SORBS1↑, RHOA↑, ACTN1↑ |
| Cardiac Hypertrophy  Signaling | 0.010 | 228 | 16 | 14 | 1 | MAP3K11↑, CALM1↑, MYL6↑, RHOC↑, PLCG1↑, HRAS↑, PPP3CC↑, EIF2B2↑, MYL9↑, GNB1↑, PLCD3↓, MYL12A↑, PLCB4↑, RHOA↑, MAPKAPK2↑, HSPB1↑ |
| Phospholipase C  Signaling | 0.01 | 243 | 16 | 14 | 0 | RELA↑, MYL6↑, CALM1↑, RHOC↑, PLCG1↑, ITGA5↑, PPP1R14A↑, HRAS↑, ARHGEF17↑, PPP3CC↑, ITGA3↑, MYL9↑, GNB1↑, PLCB4↑, MYL12A↑, RHOA↑ |
| Protein Kinase A  Signaling | 0.01 | 306 | 19 | 13 | 3 | RELA↑, YWHAH↑, MYL6↑, CALM1↑, PPP1R14A↑, PLCG1↑, PPP1R11↑, PPP3CC↑, MYL9↑, GNB1↑, PLCD3↓, MYL12A↑, PLCB4↑, NFKBIA↓, PDE7B↓, FLNA↑, RHOA↑, NFKBIB↑, PDE6D↑ |
| FAK Signaling | 0.01 | 98 | 9 | 9 | 0 | CSK↑, PLCG1↑, ITGA5↑, HRAS↑, VCL↑, ITGA3↑, TNS1↑, BCAR1↑, ACTG1↑ |
| fMLP Signaling in Neutrophils | 0.01 | 117 | 10 | 6 | 0 | GNB1↑, RELA↑, PLCB4↑, NFKBIA↓, CALM1↑, ARPC5L↑, ARPC2↑, HRAS↑, PPP3CC↑, NFKBIB↑ |
| Axonal Guidance Signaling | 0.04 | 422 | 21 | 21 | 0 | KLC1↑, PFN1↑, GLI2↑, PLXNA3↑, MYL6↑, CFL1↑, ARPC5L↑, TUBB2A↑, HRAS↑, ITGA5↑, PPP3CC↑, ITGA3↑, BCAR1↑, NCK2↑, MYL9↑, GNB1↑, PLCB4↑, MYL12A↑, TUBB6↑, ARPC2↑, RHOA↑ |
| Neuregulin Signaling | 0.04 | 95 | 8 | 6 | 2 | MYC↓, PICK1↑, PLCG1↑, ITGA5↑, HBEGF↑, HRAS↑, ITGA3↑, STAT5B↓ |
| PAK Signaling | 0.05 | 104 | 8 | 8 | 0 | NCK2↑, MYL9↑, MYL12A↑, CFL1↑, MYL6↑, ITGA5↑, HRAS↑, ITGA3↑ |
| Virus Entry via Endocytic Pathways | 0.05 | 92 | 8 | 8 | 0 | AP2M1↑, FLNA↑, PLCG1↑, ITGA5↑, HRAS↑, ITGA3↑, ACTG1↑, DNM2↑ |
