# Supplementary material for: Human breast cancer associated fibroblasts exhibit subtype specific gene expression profiles
Source: BMC Med Genomics. 2012 Sep 6;5:39. doi: 10.1186/1755-8794-5-39 (PMC3505468; doi:10.1186/1755-8794-5-39)
Supplement: Additional file 4 — Table S4.Fold changes and p-values obtained by qRT-PCR validation experiment for 6 genes found to be significantly different in either Her2+ vs ER+, Her2+ vs TNBC or ER+ vs TNBC comparison in microarrays data. FC=fold change, P=significance by t-test. Visual comparison of expression values between microarrays and qRT-PCR are presented in Figure 6. [file 1755-8794-5-39-S4.docx]

**Supplementary Table 1. Clinical Characteristics of Study Cohort**

| **ID** | **Race** | **Age at diagnosis** | **Tumor Histologic type** | **ER (%)** | **PR (%)** | **Her2** | **FISH** | **Tumor size (cm)** | **Histologic grade (1,2,3) (modified Bloom and Richarson Grading system)** | **no. of (+) axilla nodes** |
| --- | --- | --- | --- | --- | --- | --- | --- | --- | --- | --- |
| **TB0071** | **Caucasian** | **48** | **mixed ductal & lobular** | **90** | **90** | **0** | **n.a.** | **1.6** | **2** | **0** |
| **TB0075** | **Caucasian** | **35** | **ILC** | **95** | **95** | **0** | **n.a.** | **4.5** | **n.a.** | **3** |
| **TB0076** | **Caucasian** | **39** | **DCIS** | **0** | **0** | **3+** | **n.a.** | **0.1 microinvasion** | **n.a.** | **0** |
| **TB0098** | **Caucasian** | **49** | **mixed ductal & lobular** | **75** | **80** | **0** | **n.d.** | **3.2** | **2** | **2** |
| **TB117** | **African American** | **76** | **IDC** | **0** | **0** | **3+** | **n.d.** | **8.3; mulitple foci** | **3** | **8** |
| **TB120** | **African American** | **83** | **ILC** | **>90** | **30** | **0** | **n.d.** | **5.5 (~65% sclerotic with treatment effects)** | **n.a.** | **2** |
| **TB122** | **African American** | **57** | **IDC** | **0** | **0** | **3+ 50%** | **n.d.** | **3.8** | **3** | **17** |
| **TB123** | **white** | **30** | **IDC** | **0** | **0** | **0** | **n.d.** | **4.0; 2.2** | **3** | **6** |
| **TB125** | **black** | **60** | **IDC** | **0** | **0** | **0** | **neg** | **4.8** | **3** | **0** |
| **TB129** | **African American** | **32** | **IDC** | **0** | **0** | **3+** | **positive (ratio 2.5)** | **19.5** | **not done** | **not done** |
| **TB130** | **Caucasian** | **71** | **IDC** | **90** | **90** | **0** | **not done** | **5.3** | **2** | **1** |
| **TB134** | **black** | **28** | **IDC** | **0** | **0** | **0** | **NEGATIVE** | **3** | **3** | **23** |
| **TB136** | **Asian** | **38** | **DCIS** | **0** | **0** | **3+ (90%)** | **n.d.** | **5** | **n.a.** | **0** |
| **TB147** | **white** | **64** | **IDC** | **0** | **0** | **0** | **-** | **2.3** | **3** | **0** |
| **TB148** | **Caucasian** | **53** | **IDC** | **70** | **0** | **3+ 70%** | **n.d.** | **2.5** | **3** | **5** |
| **TB160** | **white** | **58** | **IDC** | **0** | **0** | **0** | **not done** | **2.6** | **2** | **0** |
| **TB162** | **black** | **44** | **IDC** | **0** | **0** | **0** | **not done** | **2.6** | **3** | **0** |
| **TB163** | **african American** | **79** | **ILC** | **+** | **+** | **0** | **not done** | **5.8** | **not done** | **16** |
| **TB164** | **black** | **47** | **IDC** | **0** | **0** | **0** | **not done** | **1.5** | **3** | **0** |
| **TB165** | **Caucasian** | **48** | **ILC** | **70** | **90** | **0** | **not done** | **9** | **not done** | **21** |
